# Glyphosate use in agricultural production: it’s contribution to global carbon dioxide emissions

**DOI:** 10.1080/21645698.2025.2594217

**Published:** 2025-12-18

**Authors:** Graham Brookes

**Affiliations:** PG Economics Ltd, Dorchester, UK

**Keywords:** Carbon dioxide equivalent emissions, conservation tillage, Glyphosate, No tillage, Plough-based tillage, reduced tillage, soil carbon sequestration

## Abstract

This paper estimates the annual global carbon dioxide equivalent (CO2e) emissions from the manufacture, distribution and farm level use of glyphosate and estimates the annual contribution of glyphosate to reducing CO2e emissions through its role in facilitating a shift in farming production practices that are tillage-based into conservation tillage systems based on reduced and no tillage. Total annual global use of glyphosate is 749.27 million kg of active ingredient, applied to 646.11 million (spray) hectares. The CO2e emission generated from the manufacture, distribution and application of glyphosate in global agriculture is annually 9.76 billion kg. The total global combined annual fuel and soil carbon retention-related CO2e emissions of conservation tillage are a net reduction of −138.2 billion kg CO2e. This compares with +41.47 billion kg CO2e emissions if this area had been plowed with conventional tillage practices. Therefore, conservation tillage practices provide a net reduction in combined annual fuel and increased soil carbon retention-related emissions of −179.67 billion kg CO2e relative to a conventional plow-based alternative production system. The total global combined annual fuel and soil carbon retention-related CO2e emissions of conservation tillage attributable to glyphosate is estimated at a net reduction of −41.93 billion kg CO2e. This compares with +13.01 billion CO2e emissions if this area had been plowed, providing a net reduction in combined fuel and soil carbon-related emissions attributable to glyphosate of −54.94 billion kg CO2e annually or the equivalent of taking 21.8 million cars off the road each year.

## Introduction

The herbicide active ingredient glyphosate is widely used in agriculture primarily for weed control across a range of crops and is a key part of crop production systems that use genetically modified herbicide tolerant (GM HT) crop technology.

It is used for controlling weeds in land preparation before crop planting: this may be part of ground clearance (e.g., for plantation crops) or to clear weeds and old crop material before planting of seasonal and field crops (e.g., corn, rice). It is also used between crop rows and surrounding field edges and bunds during crop growth (this occurs mostly in plantation crops, but also by some farmers growing field crops) and as “over the top” weed control in GM HT (tolerant to glyphosate) crops.

Its use impacts greenhouse gas (GHG) emissions through the energy and resources required in its manufacture, distribution and use by farmers as well as through its contribution in facilitating the adoption of farming practices that both reduce emissions and store carbon in soils.

The first, more straightforward part are emissions generated through the manufacture, distribution and application of glyphosate which are mostly attributed to energy use in the processes from mining of raw materials through to application by mechanical sprayer at the farm level. The second aspect relates to the possible contribution of glyphosate to reducing CO2e emissions through its role in facilitating a shift in farming production practices that are tillage-based into reduced and no tillage systems (collectively referred to as conservation tillage: Table 1) in which these tillage practices lead to CO2e reduction via additional soil carbon storage and reduced fuel use in soil preparation.

**Table 1.** Conservation tillage (cot): main practices and definitions.

Conventional tillage (CT): not a conservation tillage practice but the baseline against which COT is compared, conventionally tilled prior to planting the next crop (residue cover 0%-15%) eg, inversion tillage using a plough followed by multiple cultivation trips.
Reduced tillage (RT): full width tillage that disturbs the entire soil surface prior to planting the next crop, tillage tools such as chisel ploughs, field cultivators, rotary harrows are used, and weeds are controlled by cultivation and herbicides. With RT methods of mulch-till and ridge till, crop residue remains on the surface (this corresponds to a residue cover of 16%-30% for all crops other than maize, for which there is a reduced tillage category with a higher crop residue cover of 31%-50%).
No-till (NT): the least intensive form of tillage where a minimal amount of soil disturbance is made to ensure a good crop stand and yield. NT methods include zero-till, slot till, direct seeding and strip-till. The soil is not tilled prior to planting the next crop and substantial crop residue remains on the surface (this corresponds to a residue cover of >30% for all crops other than maize, for which the residue cover is >50%).
Permanent maintenance of a vegetative mulch cover to soil: on at least 30% of the cropping area
Rotation or sequencing of crops involving annual and perennial crops and/or sequencing involving a mix of legume and non-legume crops and cover crops

The objectives of this analysis were to firstly quantify the annual global CO2e emissions associated with the manufacture, distribution and farm level application of glyphosate and secondly, to estimate the possible annual contribution of glyphosate to reducing CO2e emissions through its role in facilitating a shift in farming production practices that are tillage-based into conservation tillage systems founded on reduced and no tillage.

## Literature Review

The extent to which conservation tillage farming systems (COT) increase the amount of organic carbon stored or sequestered in the soil has been examined in numerous papers, many of which are in peer reviewed journals. These are summarized in Appendix one and include meta-analyses.

Whilst there are some publications and meta-analyses that argue there are little or no changes in total soil organic carbon (SOC) levels associated with changes from a CT-based tillage production system to NT/RT-based systems (e.g., Hermle et al., 2008^1^, Powlson et al., 2011^2^,2014^3^), there is a significantly larger body of evidence that argues that SOC levels increase as a result of the adoption and maintenance of RT/NT systems. Some of the key sources arguing that conservation tillage systems increase SOC levels include the Intergovernmental Panel on Climate Change (IPCC, 2006^4^), Robertson et al. (2000^5^), Johnson et al. (2005^6^), Calegari et al. (2008^7^), Baker et al. (2007^8^), Angers and Eriksen-Hamel (2008^9^), Blanco-Canqui and Lal (2008),^10^ Lal (2004^11^), Lal (2005),^12^ Lal (2010^13^), Bernaccchi et al. (2005^14^), Michigan State University (2016^15^), Buragiene et al. (2019^16^), Mangalassery et al. (2014^17^), Nicoloso and Rice (2019^18^), Haruna and Nkongolo (2019^19^), Haddaway et al. (2017^20^), Hassan et al. (2022^21^), Ogle et al. (2019^22^), Ogle et al. (2023^23^), Mazzonicini et al. (2016^24^), Abdulla et al. (2016^25^), Liang B et al. (2020^26^), Corsi et al. (2012^27^), Sá et al. (2020^28^, 2024^29^ and 2025)^30^ and Reicosky and Kassam (2022^31^).

The literature highlights difficulties in estimating the precise contribution conservation tillage systems make to soil carbon sequestration levels and variations between the findings of different research finding reflect a variety of reasons. These include:

- The duration of studies: due to factors such as seasonal climate changes, land use and land management changes, 10 years is generally considered to be the minimum required;
- The depth at which soil organic carbon levels are measured – there is widespread agreement that there is a positive relationship between conservation tillage and soil organic carbon (SOC) deposition in the top 15 cm of soil, though this diminishes below 15-30 cm (Abdulla et al., 2016^25^);
- Soil type: soil type does not influence the effects of tillage on SOC stocks or concentrations from 0 to 15 cm, but does influence the effects deeper down (15-30 cm), SOC concentrations tend to be greater in sandy clay loam and silty clay soils under NT compared to tillage (Haddaway et al., 2017^20^);
- Latitude/Climate: differences in SOC between NT and tilled soils may be greater in arid rather than humid climates (Abdulla et al., 2016^25^);
- The interaction of conservation tillage with other management practices such as crop cover, crop rotation or sequencing;
- If a specific crop area is in continuous conservation tillage crop rotation, the full soil carbon sequestration (storage) benefits described in most literature can be realized. However, if the conservation tillage crop area is returned to a conventional (plow-based) tillage system, a proportion of the soil organic carbon gain will be lost. This means that carbon storage only becomes permanent when farmers adopt a continuous conservation tillage system which, in turn, is highly reliant on effective weed control (typically herbicide-based);
- Some researchers argue that level of soil organic carbon (SOC) reaches an equilibrium when the amount of carbon stored in the soil equals the amount of carbon released (the Carbon-Stock Equilibrium (CSE): e.g., Hassink, 1997^32^, Georgiou et al., 2022^33^). This hypothesis implies that as equilibrium is reached, the rate of soil carbon sequestration may decline and therefore if equilibrium is being reached after 20 to 25 years of land being in conservation tillage, the subsequent rate of soil carbon sequestration may decline. This CSE hypothesis is, however, not universally accepted and other researchers question whether applying a CSE assumption in soil models is valid because of the scope for very old soils to continue to store carbon. For example, Lal (2004)^11^ and Ussiri and Lal (2009)^34^ observed increases in SOC in the top 30 cm of soil of 1.37 Mg C ha^−1^ year^−1^ (1,370 kg of carbon (C) ha^−1^yr^1^) after 43 years of continuous maize under no tillage. This compared with a rate of 0.73 Mg C ha^−1^ year^−1^ (730 kg of C ha^−1^yr^1^) for maize grown using conventional tillage (Ussiri and Lal, 2009^34^);
- The combined effect of temperature, moisture levels, soil texture and depth also affect SOC levels. Ogle et al. (2019)^24^ identified that the depth of significant SOC additions with NT practices is highest for warm, dry, loamy, silty and clayey soils down to at 70 cm depth but only down to 5 cm for cool, dry, loamy, silty and clayey soils/tropical dry, sandy soils with the most temperature/moisture/soil texture combinations showing significant differences down to 30 cm depth. Across most of these temperature/moisture/soil type combinations, the “delta” SOC increase provided by the adoption of NT tillage estimated by this paper was within the range of 150 C ha^−1^yr^1^ to 540 kg C ha^−1^yr^1^.

It is also noteworthy that the key authority assessing and monitoring global emissions, the IPCC and several other complex national models^a^^a.^For example, the United States Department of Agriculture’s COMET-Planner^44^ applies emission reduction coefficients for changes in tillage practice from conventional tillage (CT) to no tillage (NT) and reduced tillage (RT) based on a meta-analysis of the relevant literature or the Michigan State University – US Cropland Greenhouse Gas Calculator (http://surf.kbs.msu.edu/.)^102^. There are also many subscription-based online tools that estimate farm level greenhouse gas emissions (e.g., the Cool Farm Tool in the UK^103^ which has modules covering a range of crops grown in a variety of countries). These tools all draw on the peer review literature referred to above. available to estimate the level of carbon sequestered in agricultural (cropping) production systems assume a constant rate of carbon sequestration occurs for at least up to 20 years. The IPCC, for example, assumes a twenty-year total for SOC deposition and “slices this up” into 20 equal parts for quantification over a 20-year period.

Some research has also suggested that NT- based system adoption may lead to increases in nitrous oxide (N_2_O) emissions and hence result in the overall carbon footprint of NT systems being negative (Don and Jantz, 2013^35^). This assertion was assessed in detail in a meta-analysis by Freitag et al. (2024^36^ which reviewed 88 papers, 50 of which were examined in the Don and Jantz paper. Freitag et al. review of this literature concluded that “*overall the claim by Don and Jantz, 2013 was not confirmed by this literature review. On the contrary, it seems that the long-term absence of tillage, together with permanent soil biomass cover and diverse crop vegetation, can reduce N*_*2*_*O emissions”*. Analysis by Karki et al. (2025)^37^ of 90 nitrous oxide observations also concluded that conservation tillage agriculture with cover crops does not affect nitrous oxide emissions.

The use of fossil fuel energy for agricultural machinery in NT and RT systems also contributes further to reductions in CO2e emissions with NT/RT production-based systems using less fuel for soil preparation than CT-based systems of production. Specific research at a regional or country level that confirms this includes Australia (Maraseni and Cockfield, 2011^38^), Spain (Hernanz et al., 2014^39^ and Marguez et al., 2011^40^), Slovenia (Stajnko et al., 2009)^41^ and the USA (Conservation Effects Assessment Project: CEAP, 2016^42^). For example, the CEAP-Crop Conservation Insight report for 2016 estimated that annually in the USA the widespread adoption of conservation tillage has resulted in a fuel use reduction of 3,075.3 million liters of diesel equivalents, within which the application of NT accounted for 72% of the total reduction in fuel use.

Complex national models such as the USDA’s Energy estimator for Tillage Model^43^ and The Voluntary Reporting of Greenhouse Gases-Carbon Management Evaluation Tool (COMET-VR^44^ that estimate the level of carbon sequestered in agricultural (cropping) production systems also assume that NT/RT-based systems use less fuel for tillage than CT-based systems. For example, the Voluntary Reporting of Greenhouse Gases-Carbon Management Evaluation Tool (COMET-VR^44^ uses a reduction of 41.8 litres/ha when CT is replaced by NT on non-irrigated maize and a reduction of 59.7 litres/ha in the case of soybeans in Nebraska.

Moving onto glyphosate’s influence in facilitating the adoption and maintenance of conservation tillage practices, this primarily stems from its part in providing an effective form of weed control, especially in the land preparation phase before field crops like cereals and oilseeds are planted. This is particularly important in relation to GM HT (tolerance to glyphosate) based production systems but is also of relevance to conventional production systems.

The importance of GM HT (tolerant to glyphosate) technology in the adoption of NT/RT systems was first highlighted by the American Soybean Association (ASA: 2001)^45^ and CTIC (2002^46^). The ASA study, for example, found that the availability of GM HT soybeans facilitated and encouraged farmers to implement reduced tillage practices; most growers surveyed indicated that GM HT soybean technology had been the factor of greatest influence in their adoption of reduced tillage practices. Fernandez-Cornejo et al. (2012)^47^ also concluded over an eleven-year period (1996–2006) that GM HT soybean adoption had led to a significant increase in the adoption of conservation tillage (RT/NT) with a one percent increase in GM HT soybean adoption leading to a 0.21% increase in conservation tillage. Similarly, Finger et al. (2009^48:^ based on a survey of Argentine soybean growers) identified that the combination of herbicide tolerance and NT were the key drivers to adoption of GM HT soybeans, facilitating easier crop management and reducing herbicide costs. In addition, Perry et al. (2016)^49^ reviewed 51 papers examining the evidence for a relationship (complementarity) between the adoption and use of glyphosate tolerant crops and conservation tillage practices. It concluded that the evidence in favor of complementarity between glyphosate tolerant crops and conservation tillage outweighs the evidence against it. The research further examined the relationship using farm level data from the US for nearly 30,000 soybean farmers over the period 1998–2011 and confirmed complementarity, with adoption rates for CT and NT estimated to have been 10% and 20% respectively higher than would otherwise have occurred in the absence of glyphosate tolerant soybean technology. Other analysis identifying correlations between glyphosate use, GM HT crops and carbon sequestration include Sunderland et al. (2021)^50^ relating to Canada and Rodriguez et al. (2021)^51^ relating to Argentina.

There is also broad consensus that glyphosate plays an important role in facilitating the adoption and operation of conservation tillage systems in conventional cropping systems (Wynn and Webb, 2022^52^, Walsh and Kingwell, 2021^53^, Loureiro et al., 2019^54^, Page et al., 2020^55^, Prado, 2021^56^, Brookes, 2019^57^).

In Europe, where conservation tillage usage is solely in conventional cropping systems and less widespread than in North and South America, the widespread use of glyphosate reflects its broad spectrum of activity and relative low number and incidence of weed resistance. This is related to the widespread resistance of some key weed species like blackgrass (*Alopecurus myosuroides*) to many selective herbicides with different modes of action which has left limited chemical control alternatives for the control of this highly competitive weed (Moss, 2017^58^).

As glyphosate is rarely used in isolation for weed control in the land preparation and between crop period, key to facilitating conservation tillage production systems in both GM HT and conventional crops, only a proportion of all greenhouse gas emission savings associated with the application of conservation tillage practices can be reasonably attributed to glyphosate use.

Nevertheless, whilst conservation tillage can be practiced from a technical perspective without the use of glyphosate and/or other herbicides, the research evidence discussed above and data available on conservation tillage areas, crops typically grown within the system (largely cereals, oilseeds, pulses) and the area of these crops regularly receiving applications of herbicides and glyphosate, shows that the vast majority of crops grown under conservation tillage use herbicides as the primary form of weed control and within this, glyphosate is the most applied herbicide. Farmer survey-based research about the reasons for the dominance of herbicide use and within this, a preference for glyphosate being in the mix of active ingredients used (e.g., Brookes, 2019^57^, Wynn and Webb, 2022^52^, Walsh and Kingwell, 2021^53^, ASA, 2001^45^, CTIC, 2002^46^ Sunderland et al., 2021)^50^ show a consistent response. The high prevalence of herbicide-based weed control programs featuring glyphosate in conservation tillage agriculture reflects a combination of the weed control efficacy provided allied to cost effectiveness. Much of the same cited research above also shows that when questioned about how farmers might change their production practices if they no longer had access to glyphosate, a consistent response was that where conservation tillage production was practiced it was likely to be dropped with reversion to a plow-based system.

The literature review also identified that crops typically grown within conservation tillage systems are largely cereals, oilseeds and pulses and almost all crops grown under conservation tillage use herbicides as the primary form of weed control. Within this, glyphosate is the most applied herbicide (Brookes, 2019^57^, Wynn and Webb, 2022^52^, Walsh and Kingwell, 2021^55^, ASA, 2001^45^, CTIC, 2002^46^ Sunderland et al., 2021^50^).

As well as glyphosate use and its role in the adoption of conservation tillage practices facilitating the reductions in greenhouse gas emissions via reduced fuel use and additional soil carbon sequestration, it may also affect GHG emissions via changes to yields and crop profitability. Estimating the possible GHG emissions changes associated with yield changes or impacts on the relative profitability of different crops (which influences planting decisions) is, however, difficult due to the complex array of variables that affect them. Examination of these “knock-on” issues would add considerably to the research requirement and length of any subsequent paper. As such, no estimates are provided in this report, which focuses on the core assessment of the direct impact of glyphosate use on GHG emissions, inclusive of its role in facilitating conservation tillage practices.

Nevertheless, in furthering the debate about the potential GHG emission impacts associated with the use of glyphosate via knock on effects associated with yield changes and crop production mixes, several effects could arise. Glyphosate use may lead to improved weed control which in turn leads to higher yielding crops (e.g., as experienced with the adoption of GM HT technology in soybeans in Romania, maize in Vietnam and the Philippines: Brookes, 2022a^59^). Higher yielding crops assimilate more carbon dioxide into carbohydrate, oxygen and water than lower yielding crops (Simpkin et al., 2019^60^). Increasing crop yields can also result in an increase in carbon inputs from crop residues into soils which have a positive effect on soil carbon stocks (Berntsen et al., 2006^61^).

Improved yields and additional production from second cropping (e.g., of soybeans in South America after the introduction of GM HT technology in the mid-1990s: Brookes, 2022a^59^ effectively “replaces” the need to extend crop production into new lands (which will require the switching of land uses from other crops, grazing land and/or non-agricultural land converted into cropping of soybeans, maize, cotton and canola). Where this land that would otherwise have been brought into agriculture remains in alternative uses that sequester important levels of GHGs (e.g. forestry), it is likely that the net effect on GHG emissions is positive. Intensification of production is also crucial if new land is not to be brought into production. For example, analysis by Tilman et al. (2011^62^ into meeting projected global food demand by 2050 suggests that moderate intensification delivers significant (three-fold) greenhouse gas emission savings compared to a scenario of no additional intensification.

## Methodology

### Quantification of Global Glyphosate Use

To provide a baseline for estimating the annual CO2e emissions associated with the use of glyphosate, data on the use of glyphosate globally was collected and analyzed. Data was compiled from a combination of sources including nationally published sources (e.g., United States Department of Agriculture National Agricultural Statistical Service: USDA NASS (in the USA), private market research and subscription service providers like Kynetec (and formerly from Kleffmann), trade data, the author’s own estimates, as well as additional supporting literature.

The baseline used for the analysis was the annual usage of glyphosate in global agriculture based on the average annual global use of glyphosate across the most recent four years for which comprehensive data was available (2019–2022). This means that the CO2e emissions calculated for the manufacture, production, distribution and application of glyphosate directly relate to the average annual usage in the period 2019–2022.

### Calculation of Emissions from Glyphosate Manufacture, Distribution and Farm Level Use

There is a very limited literature available that examines the CO2e emissions from the activities of manufacturing and distributing pesticides to farmers. Where papers have been identified, they are not recent, for example Audsley et al. (2009^63^), which itself draws on data going back into the 1990s.

The most current and detailed source available is EcoInvent^64^, the database of lifecycle inventory data. This dataset is widely used by businesses around the world to assist with calculations of emissions that are required to fulfill legal environmental reporting requirements. The dataset is also utilized by the International Panel on Climate Change (IPCC). The carbon dioxide generation from the manufacture and distribution of glyphosate from this source is 11.2 kg per kg of active ingredient (Ecoinvent^60^, accessed March 2025)

Emissions associated with the application of glyphosate arise from fuel use in application where mechanical forms of application are used. The fuel used in a typical method of mechanical application is with a 90-foot boom sprayer. Literature examining the fuel used for the application of pesticides, including herbicides, was reviewed including periodically updated national and regional farmer management and extension type guides around the world. These sources all broadly show a consistent narrow range of fuel usage of between 0.5 liters per hectare and 1 liter per hectare. For example, Bowling J (2008)^65^ found (in Australia) a range of 0.5 to 1.01 liters per ha. The core analysis in this paper uses 0.84 liters/ha drawn from Lazarus (2019)^66^ that relates to the USA although sensitivity analysis is also presented to the full range of 0.5 liters/ha to 1 liter/ha.

Based on this, in terms of GHG emissions, each liter of tractor diesel consumed contributes an estimated 2.67 kg of carbon dioxide into the atmosphere (one application emits 2.24 kg/ha carbon dioxide within a range of 1.335 kg/ha if 0.5 litres/ha is the assumed fuel use to 2.67 kg/ha if 1 liter of fuel is the assumed fuel use). As some farmers, mostly in developing countries, apply agrochemicals by hand and do not use any fuel during application, this factor has been taken into consideration in the analysis by reducing the total spray area of glyphosate to which the fuel-related emissions have been assumed to have been applied. For example, for India, the application fuel use and associated emissions have been applied to only 15% of the total glyphosate spray area, reflecting the limited use of mechanical spray application in a country where the average size of farm growing cereals and oilseeds is under 1 hectare.

### Literature Review

A primary aim of the literature review was to identify the evidence about emissions after adoption of conservation tillage practices, soil organic carbon levels and other possible emissions such as nitrous oxide (N_2_O) relative to conventional tillage practices. This review (see Introduction, specific Literature Review section, Appendix 1 and Supplementary material) provided the evidence for setting assumptions for calculating emission savings associated with the adoption of conservation tillage practices (see below). In addition, the literature review provided the evidence for confirming the facilitating role of glyphosate in the adoption and maintenance of NT/RT-based conservation tillage practices.

### Calculation of Emissions Reduction from the Use of Conservation Tillage

The fuel use assumptions for conservation tillage practices used in this paper are drawn from a review of literature, in particular the USDA’s Conservation Effects Assessment Project (CEAP: 2016^42^), CTIC (2002^46^), the USDA Energy Estimator for Tillage Model (2013^43^ and the online USDA Comet-VR model (2014^44^). In addition, country-specific data such as for Australia are drawn from relevant literature (Maraseni T and Cockfield G, 2011^38^). These are detailed in Table 2.

**Table 2.** Liters fuel per ha and equivalent Co2e emissions (in brackets).

	No till	Reduced till	Conventional till
USA	16.83 (44.94)	21.57 (57.59)	50.51 (134.86)
Australia	13.70 (36.58)	Not available	27.60 (73.69)
All other countries	21.89 (58.45)	38.62 (103.11)	49.01 (130.86)

Notes.

1. RT rate is the weighted (by area) average of the classifications ‘continuous mulch, seasonal no till and continuous no till, classified broadly as Conservation tillage total by CEAP.

2. No till refers to continuous no till (CEAP definition).

3. Conventional tillage refers to continuous plow-based tillage (CEAP definition).

The soil carbon storage assumptions are also drawn from the literature review and complex models cited. The analysis presented in the results section uses assumptions that country-specific, where available, and in the absence of these draws on conservative values that take into consideration the finding of assumptions drawn from meta-analyses. These are as follows:

*USA*:.US-specific papers on soil carbon storage with conservation tillage identified a range of estimated values. These include 100 kg C ha^−1^yr^−1^ to 500 kg C ha^−1^yr^−1^ range (Kassam et al., 2019^67^), 570 C ha^−1^yr^−1 (^West and Post, 2002^68^), 240 C ha^−1^yr^−1^ to 400 C ha^−1^yr^−1^ (Lal 1999^69^ and 230 C ha^−1^yr^1^ to 320 C ha^−1^yr^1^ (Ogle, 2019^22^). For this analysis, the US-specific assumption for the difference between NT and CT tillage has been 275 C ha^−1^yr^−1^ based on NT systems store 250 kg C ha^−1^yr^1^ compared to CT systems that emit 25 kg C ha^−1^yr^1^

*Argentina, Brazil and other countries of South America*: The soil carbon retention assumption used is 175 kg C ha^−1^yr^1^ for NT soybean cropping and CT systems release 25 kg C ha^−1^yr^1^ (a difference of 200 kg C ha^−1^yr^1^). This is a conservative estimate based on Alvarez et al. (2014^70^).

*Canada*: The soil carbon retention assumption used is NT systems store 211 kg C ha^−1^yr^1^ compared to CT systems that emit 10 kg of C ha^−1^yr^1^ based on Liang B (2020^26^)

*Italy*: The soil carbon retention assumption used is NT systems store 132 kg of C ha^−1^yr^1^ compared to CT systems that emit 6 kg C ha^−1^yr^1^ (Mazzonicini et al., 2016^22^).

*South Africa*: The soil carbon retention assumption used is NT systems store 125 kg C ha^−1^yr^1^ compared to CT systems that emit 25 kg C ha^−1^yr^1^ (based on Nyambo P, Cornelius C and Araya T, 2020^71^)

*Generic/general (not country specific as estimated based on analysis of data from several countries):*

Some of the meta-analysis type studies include:

*IPCC (2006* ^4^): all conservation tillage (NT and RT) in North America was estimated to add to SOC levels within a range of 50 C ha^−1^yr^1^ to 1,300 kg C ha^−1^yr^1^ (it varies by soil type, cropping system and eco-region), with a mean of 300 kg C ha^−1^yr^1^;

*Haddaway et al. (2017* ^20^). In the upper soil layer (up to 30 cm depth) the NT versus CT difference was equal to 460.6 kg C ha^−1^yr^1^ (drawn from 351 studies)

*Ogle et al., 2019* ^22^. Across most of these temperature/moisture/soil type combinations, the “delta” SOC increase provided by the adoption of NT tillage estimated in this paper was within the range of 150 C ha^−1^yr^1^ to 540 kg C ha^−1^yr^1^.

Given the range of possible impacts identified in meta studies, the analysis uses values at the conservative end of the range for all countries where no country-specific analysis is available. Thus, the general values used are those referred to above for South American countries, soil carbon retention of 175 kg C ha^−1^yr^1^ for NT soybean cropping and CT systems release 25 kg C ha^−1^yr^1^ are used for all other countries.

The baseline global area of land using conservation tillage practices used in the analysis is 202.8 million hectares. The primary source for this is Kassam et al., 2022^72^, https://www.ca-global.net/ca-stat), although other sources, including FAO, CEAP (USA), Eurostat). The years from which this dataset relates vary according to differences in the availability of data by country. As the baseline year for conservation tillage areas used in the most comprehensive data source, Kassam et al., 2022^72^ is 2018/19, this is the baseline used, except for the USA, where the average annual continuous NT area recorded by the latest CEAP survey for the 2013–2016 was used.

### Calculation of Contribution of Glyphosate to Emissions Reduction from the Use of Conservation Tillage

Calculating the contribution of glyphosate to emissions reductions associated with conservation tillage involved the following stages:

- Firstly, identification of evidence of a link between glyphosate use and adoption/maintenance of NT/RT-based conservation tillage practices;
- Establishment of a methodology for estimating the contribution of glyphosate use to annual CO2e emissions associated with conservation tillage practices

The literature review provided the first step to identify any link between glyphosate use and adoption/maintenance of NT/RT-based conservation tillage. This review, as discussed in the introduction and earlier specific literature review section, confirmed that glyphosate plays an important role in facilitating the adoption and maintenance of conservation tillage practices. This is particularly important in relation to GM HT (tolerance to glyphosate) based production systems but is also of relevance to conventional production systems.

Based on the literature review, the methodology applied to estimate a contribution of glyphosate share to GHG savings associated with the adoption and use of NT/RT-based conservation tillage practices was as follows:

### For Conventional Crops

- Identification and quantification of all herbicides used (in terms of spray areas) for weed control in the pre-plant/between successive crops and pre-emergence phase in the main crops typically grown under conservation tillage systems (primarily cereals and oilseeds) by country;
- Within this dataset, identification and quantification of the specific spray area of glyphosate used in these phases of production for the main crops in which conservation tillage are typically used, by country.

### For GM HT Crops (Tolerant to Glyphosate)

- The same approach was applied to conventional crops – based on the (spray area) share of glyphosate relative to other herbicides in the pre-plant/between crops and pre-emergence phase;
- Identified the share of total GM HT crops solely tolerant to glyphosate relative to GM HT crops tolerant based on estimates of GM HT trait-specific seed sales in the crops where GM HT are used (corn, cotton, canola and soybeans).

## Results

The sub-sections below detail the results calculated in the research, drawing on the datasets and assumptions referred to in the methodology section. To further assist readers in following how these calculations have been made, additional detailed information that takes readers through the estimates for one country (Argentina) has been provided in Appendix 3.

## Glyphosate Use

### Total Global Glyphosate Use

Worldwide annual herbicide use, based on an annual average of usage over the period 2019 to 2022 is about 1,690 million kg of active ingredient applied to about 2,929 million hectares (spray hectares, not crop hectares). Within this, total annual global use of glyphosate was 749.27 million kg of active ingredient, which was applied to 646.11 million hectares (spray hectares). Therefore, in terms of total global herbicide use, glyphosate accounts for about 44% of the total volume of herbicide active ingredient use and 22% of the total herbicide spray area.

The main country-level users of glyphosate are Brazil, the USA and Argentina, followed by China, South Africa, Canada, Australia, the European Union, Paraguay and India (Table 3).

**Table 3.** Glyphosate use: by country (2019–2022 annual averages).

	‘000 kg active ingredient	‘000 ha (spray area)
Argentina	116,137	125,345
Australia	27,723	38,767
Brazil	163,341	124,699
Canada	31,469	35,536
China	66,685	41,351
EU	20,980	25,844
India	11,988	12,276
Indonesia	11,239	10,984
Kazakhstan	5,245	9,692
Paraguay	13,487	8,399
Russia	10,490	9,692
South Africa	50,950	7,107
USA	125,128	115,654
Others	94,408	80,764
**Total**	**749,270**	**646,110**

Sources: USDA NASS, private market research companies (e.g., Kynetec), industry estimates, national import/export statistics, author own calculations.

Note: the category of others includes a total of 54 countries, each of which accounted for 1% or less of total usage.

In terms of the glyphosate spray area, the largest users are also Argentina, Brazil and the USA, followed by China, Australia, Canada, the EU, India, Indonesia, Russia, Kazakhstan, Paraguay and South Africa (Table 3).

By crop use, Table 4 shows that the main crops in which glyphosate use occurs are soybeans and corn which account for 30% and 18% respectively of total use. The other main user sectors are wheat, fallow, forage crops, plantation crops and cotton.

**Table 4.** Annual average glyphosate usage 2019–2022 by crop/use.

Crop	Active ingredient use (‘000 kg)	Spray area (‘000 ha)
Soybeans	227,534	192,674
Corn	136,167	113,082
Wheat	46,895	59,848
Fallow	42,889	36,340
Forage crops	22,762	28,617
Canola	13,871	15,864
Plantation crops	37,266	29,466
Rice	20,641	16,435
Cotton	26,719	23,899
Sugar cane	10,788	7,453
Other crops and uses	163, 739	122,430
**Total**	**749,270**	**646,110**

Sources: USDA NASS, private market research companies (eg, Kynetec), industry estimates, national import/export statistics, own calculations.

## CO2e Emissions from the Manufacture and Supply/Distribution of Glyphosate to Farmers

As discussed in the methodology section, the carbon dioxide generation from the manufacture and distribution of glyphosate is 11.2 kg per kg of active ingredient (Ecoinvent^60^). Applying this value to the annual volume of glyphosate use (749.27 million kg of active ingredient), the total amount of CO2e emissions associated with the annual manufacture and distribution of glyphosate used in global agriculture is 8.39 billion kg. A breakdown of these emissions by country where the glyphosate is distributed to for use is provided in Table 5 and a more detailed breakdown for all countries is available in Appendix 2.

**Table 5.** Annual average CO2e emissions from the manufacture and distribution of glyphosate used in global agriculture by country of use: baseline 8.39 billion kg.

	Million kg CO2e	%
Argentina	1,304.5	15.5
Australia	309.3	3.7
Brazil	1,831.6	21.8
Canada	351.0	4.2
China	744.9	8.9
EU	234.9	2.8
India	125.8	1.5
Indonesia	125.8	1.5
Kazakhstan	58.7	0.7
Paraguay	117.5	1.4
South Africa	553.7	6.6
USA	1,397.5	16.7
Others	1,234.8	14.7
**Total**	**8,390**	**100**

Note: The emissions from the manufacture and distribution of glyphosate stated for each country are based on applying the global average 11.2 kg of CO2e emissions per kg active ingredient of glyphosate manufactured and distributed to farm level (source: Ecoinvent) to the total amount of glyphosate active ingredient used in agriculture in each country. Glyphosate manufacture does not take place in each country and a significant volume of glyphosate is traded between countries. The emissions values from the manufacture and distribution per country therefore reflect farm level usage not where the manufacture of glyphosate takes place.

These estimates exclude emissions associated with the formulation of products that contain glyphosate. Emissions associated with formulations vary by formulation and the author is not aware of any source that adds this level of detail. It is likely that the emissions associated with formulation are small relative to the 11.2 kg CO2e calculated by the Eco Invent resource.

## CO2e Emissions from the Application of Glyphosate

Based on the assumption that 0.84 liters of fuel are used per hectare sprayed with glyphosate (and its equivalent value of 2.24 kg carbon dioxide emitted per hectare of crop sprayed), and applying this to the annual volume of glyphosate used in global agriculture, the total annual amount of CO2e emissions associated with the application of glyphosate by farmers used in global agriculture is 1.37 billion kg. A full breakdown by country is provided in Appendix 2.

If the CO2e emissions are calculated around the main range of fuel used to apply herbicides around the world (0.5 to 1 liter of fuel per spray ha), the total annual CO2e emissions associated with the application of glyphosate are within a range of 0.82 billion kg and 1.63 billion kg.

## CO2e Emissions from the Use of Conservation Tillage

The analysis presented below uses the fuel use and soil carbon storage assumptions drawn from the literature review applied to the estimated global area using conservation tillage (201 million ha: Sources Kassam et al., 2022^72^, CEAP, 2016^42^).

The total annual fuel-related CO2e emissions associated with NT/RT-based conservation tillage operations is 10.44 billion kg CO2e emissions (Table 6). This compares with 23.54 billion kg CO2e emissions if this area had been plowed using CT, providing a net reduction in fuel-related emission of 13.1 billion kg CO2e emissions (−55.6%).

**Table 6.** Annual global fuel use and CO2e emissions for NT/RT-based conservation tillage area compared to levels if the same area was tilled by plough: by main country.

Country	NT/RT fuel use (million litres)	NT/RT fuel use CO2e emissions (million kg)	CT fuel use million litres)	CT fuel use CO2e emissions if this area had been ploughed (million kg)	Net NT/RT fuel use related emission savings relative to CT tillage (CO2e million kg)
Argentina	720.4	1,923.4	1,612.8	4,306.2	−2,382.8
Australia	306.1	817.2	632.8	1,689.5	−872.3
Bolivia	40.6	108.3	90.8	242.5	−134.0
Brazil	941.3	2,513.3	2,107.5	5,627.0	−3,113.7
Canada	475.9	1,270.6	1,065.5	2,844.8	−1,574.2
China	197.0	526.0	441.1	1,177.7	−651.7
EU	65.8	175.7	147.3	393.3	−217.6
India	76.6	204.6	171.5	458.0	−253.4
Kazakhstan	65.6	175.3	147.0	392.6	−217.3
Mexico	3.6	8.7	7.3	19.5	−10.8
Paraguay	69.1	184.6	154.8	413.3	−228.7
Russia	100.0	266.9	223.8	597.5	−330.6
South Africa	35.2	93.9	78.8	210.3	−116.4
Ukraine	19.7	52.6	44.2	117.8	−65.2
UK	12.3	32.8	27.5	73.5	−40.7
Uruguay	28.0	74.7	62.7	167.3	−92.6
USA	702.5	1,875.8	1,687.4	4,505.5	−2,628.7
Others	50.9	135.8	113.9	304.0	−168.2
**Total**	**3,912.2**	**10,440.5**	**8,816.2**	**23,539.3**	**-13098.9**

The total annual soil carbon storage/sequestered associated with the conservation tillage area was −148.64 billion kg CO2e. This compares with +17.93 billion kg CO2e emissions if this area had been plowed, providing a net reduction in soil carbon storage-related emissions of −166.53 billion kg CO2e (Table 7).

**Table 7.** Annual global soil carbon retention and CO2e emissions reduction for NT/RT-based conservation tillage area compared to levels if the same area was tilled by plough: by main country.

Country	NT/RT soil carbon retention (CO2e emissions equivalent billion kg)	CT soil carbon retention (CO2e emissions equivalent billion kg)	Total NT/RT soil carbon retention relative to CT alternative (CO2e billion kg)
Argentina	−21.13	+3.02	−24.32
Australia	−14.72	+2.10	−16.82
Bolivia	−1.19	+0.17	−1.36
Brazil	−27.62	+3.95	−31.57
Canada	−16.83	+0.80	−17.63
China	−7.63	+0.83	−8.46
EU	−4.41	+0.72	−5.13
India	−2.25	+0.46	−2.71
Kazakhstan	−4.40	+0.39	−4.79
Mexico	−0.96	+0.02	−0.98
Paraguay	−2.03	+0.41	−2.44
Russia	−2.93	+0.60	−3.53
South Africa	−1.03	+0.21	−1.24
Ukraine	−0.58	+0.12	−0.7
UK	−0.83	+0.07	−0.9
Uruguay	−0.82	+0.12	−0.94
USA	−38.28	+3.83	−42.11
Others	−1.86	+0.28	−2.14
**Total**	**−148.6**	**+17.93**	**−166.53**

Table 6 and Table 7 also show that the main grain and oilseed producing and exporting countries of North and South America dominate CO2e emission reductions associated with the use of conservation tillage practices, accounting for over 70% of the total. These countries (USA, Canada, Argentina, Bolivia, Brazil, Paraguay and Uruguay) are also countries where GM HT crop technology has dominated production systems for more than 20 years.

The total global combined annual fuel and soil carbon retention-related CO2e emissions of conservation tillage (Table 6 and Table 7) is a net reduction of −138.2 billion kg CO2e. This compares with +41.47 billion kg CO2e emissions if this area had been plowed, providing a net reduction in combined annual fuel and soil carbon retention-related emissions of −179.67 billion kg CO2e.

## Glyphosate Use in the Land Preparation and Between Crop Period, Key to Facilitating Conservation Tillage Production Systems in Both GM HT and Conventional Crops

The methodology applied to estimate the contribution of glyphosate share to GHG savings associated with the adoption and use of NT/RT-based conservation tillage practices is based on identification and quantification of all herbicides used (in terms of spray areas) for weed control in the pre-plant/between successive crops and pre-emergence phases of production in the main crops typically grown under conservation tillage systems (primarily cereals and oilseeds) at the country level. This identified that about 35% of total usage (by active ingredient volume) is in the pre plant and pre-emergence phases, 32% is post emergent and harvest phase and 33% is classified as “undefined” use. Most of the undefined use relates to use in countries like India and China where use data to this level of disaggregation is limited.

Analysis of all herbicides used and specifically glyphosate use in the crops and categories of use/stage of crop development of most relevance to conservation tillage (Table 8) shows:

- In relation to total herbicide use, the total amount of herbicide active ingredient used in the relevant categories of use/stages of crop development for conservation tillage^b^^b.^Classified as pre plant, pre-emergent, post-harvest, soil uses and undefined use (in countries where undefined use accounts for significant shares of usage, this usage is mostly in cereals and oilseeds, the crops most grown with conservation tillage practices). is 1,143.4 million kg, equal to 68% of total herbicide use (in relation to area sprayed, it is 58% of the total spray area, equal to 1,713 million ha);
- Further focusing on the main crops in which conservation tillage is practiced (largely cereals, including rice, oilseeds and “fallow”), total annual herbicide use is 534.6 million kg (32% of total herbicide active ingredient use) on 977.1 million spray hectares (33% of the total herbicide spray area);
- Glyphosate use in the relevant stages of (crop) development in crops most grown under conservation tillage is annually 237.7 million kg of active ingredient use and 213.5 million spray hectares. This is equivalent to 44% of total herbicide active ingredient use and 21.8% of the total spray area on crops most grown under conservation tillage.

**Table 8.** Glyphosate use in stages of crop growth key to the adoption of conservation tillage: 2019–2022 annual averages.

	Active ingredient use (‘000 kg)	Spray area (‘000 ha)
Total all uses/crops	749,270	646,110
Of which		
Glyphosate use in growth stages of crops most grown under conservation tillage	237,722	213,473

Sources: USDA NASS, private market research companies (e.g., Kynetec), industry estimates, national import/export statistics, own calculations.

Note: Crops examined as “most grown under conservation tillage” cereals, oilseeds and pulses.

Table 9 provides a breakdown of this glyphosate use by country, with nearly three-quarters of total use being in North and South American countries which have the highest levels of conservation tillage adoption and where GM HT tolerant crops (notably corn and soybeans) dominate production systems.

**Table 9.** Glyphosate use in stages of crop growth key to the adoption of conservation tillage: by country (2019–2022 annual averages).

	‘000 kg active ingredient	% of all herbicides active ingredient used in these stages accounted for by glyphosate	‘000 ha (spray area)	% of all herbicides spray area in these stages accounted for by glyphosate
Argentina	43,619	55	34,598	29
Australia	7,667	47	10,438	30
Bolivia	931	76	898	52
Brazil	70,124	74	54,724	46
Canada	15,683	56	17,904	38
China	14,749	20	9,710	6
EU	4,401	31	8,200	18
India	1,964	21	2,431	5
Kazakhstan	5,165	78	9,893	23
Mexico	1,443	55	1,402	30
Paraguay	4,287	61	3,919	34
Russia	3,394	90	3,312	69
South Africa	5,954	56	4,797	28
UK	928	43	1,101	30
Uruguay	2,026	65	1,513	32
USA	40,855	39	38,193	23
Others	14,532	10	10,440	4
**Total**	**237,722**	**32**	**213,473**	**33**

Sources: USDA NASS, private market research companies (e.g., Kynetec), industry estimates, national import/export statistics, own calculations.

Note: Crops examined as ‘most grown under conservation tillage’ cereals, oilseeds and pulses.

## CO2e Emission Removal Savings Associated with Soil Carbon Sequestration with Conservation Tillage Practices Attributable to Glyphosate

The results presented below are based on applying the country-specific shares of glyphosate (in terms of spray area) in total herbicide use in the crops and categories of use/stage of crop development of most relevance to conservation tillage (Table 8 and Table 9) to the fuel use and soil carbon storage values identified above (see Table 7).

The total annual fuel-related CO2e emissions associated with conservation tillage operations attributable to glyphosate is +3.37 billion kg CO2e emissions (Table 10). This compares with +7.58 billion kg CO2e emissions if this area had been plowed attributable to glyphosate, providing a net reduction in fuel-related emission attributable to glyphosate of −4.21 billion kg CO2e emissions.

**Table 10.** Annual global fuel use and CO2e emissions for NT/RT-based conservation tillage area compared to levels if the same area was tilled by plough attributable to glyphosate: by main country.

Country	NT/RT fuel use CO2e emissions (million kg)	NT/RT fuel use CO2e emissions attributable to glyphosate (million kg)	CT fuel use CO2e emissions if this area had been ploughed ((CO2e million kg)	Net NT/RT- fuel use related emissions attributable to glyphosate (CO2e million kg)
Argentina	1,923.4	558.4	1,250.2	−691.8
Australia	817.2	246.4	509.4	−263.0
Bolivia	108.3	56.4	126.2	−69.8
Brazil	2,513.3	1,153.8	2,583.2	−1,429.4
Canada	1,270.6	482.0	1,079.0	−597.1
China	526.0	30.1	67.3	−37.3
EU	175.7	31.6	70.8	−39.2
India	204.6	9.3	20.8	−11.5
Kazakhstan	175.3	40.4	90.4	−50.0
Mexico	8.7	2.6	5.8	−3.2
Paraguay	184.6	62.3	139.6	−77.2
Russia	266.9	183.0	409.8	−226.8
South Africa	93.9	26.5	59.4	−32.9
Ukraine	52.6	11.9	26.7	−14.8
UK	32.8	9.9	22.2	−12.3
Uruguay	74.7	24.3	54.3	−30.1
USA	1,875.8	440.3	1,057.2	−617.0
Others	135.8	5.4	12.2	−6.7
**Total**	**10,440.5**	**3,374.7**	**7,584.6**	**-4,210.0**

The total annual soil carbon storage/sequestered associated with the conservation tillage area attributable to glyphosate was −45.3 billion kg CO2e. This compares with +5.4 billion kg CO2e emissions if this area had been plowed, providing a net reduction in soil carbon storage-related emissions attributable to glyphosate of −50.73 billion kg CO2e (Table 11).

**Table 11.** Annual global soil carbon retention CO2e emissions for NT-based conservation tillage area compared to levels if the same area was tilled by plough attributable to glyphosate: by main country.

Country	NT/RT soil carbon retention (CO2e emissions equivalent billion kg) from Table 7	NT/RT soil carbon sequestration CO2e emissions attributable to glyphosate (billion kg)	CT soil carbon retention (CO2e emissions equivalent (CO2e billion kg)	Net NT/RT-soil carbon sequestration CO2e missions attributable to glyphosate (CO2e billion kg)
Argentina	−21.13	−6.14	+0.87	−7.01
Australia	−14.72	−4.44	+0.63	−5.07
Bolivia	−1.19	−0.62	+0.09	−0.71
Brazil	−27.62	−12.68	+1.81	−14.49
Canada	−16.83	−6.38	+0.31	−6.69
China	−7.63	−0.44	+0.04	−0.48
EU	−4.41	−0.79	+0.13	−0.92
India	−2.25	−0.10	+0.02	−0.12
Kazakhstan	−4.40	−1.01	+0.17	−1.18
Mexico	−0.96	−0.028	+0.004	−0.032
Paraguay	−2.03	−0.68	+0.01	−0.78
Russia	−2.93	−2.01	+0.29	−2.30
South Africa	−1.03	−0.29	+0.04	−0.33
Ukraine	−0.58	−0.13	+0.02	−0.15
UK	−0.83	−0.25	+0.04	−0.29
Uruguay	−0.82	−0.27	0.03	−0.30
USA	−38.28	−8.98	+0.9	−9.88
Others	−1.86	−0.07	+0.02	−0.09
**Total**	**−148.6**	**−45.308**	**+5.424**	**−50.732**

The total global combined annual fuel and soil carbon retention-related CO2e emissions of conservation tillage attributable to glyphosate (Table 10 and Table 11) is a net reduction of −41.93 billion kg CO2e. This compares with +13.01 billion CO2e emissions if this area had been plowed, providing a net reduction in combined fuel and soil carbon-related emissions attributable to glyphosate of −54.94 billion kg CO2e. In terms of the net reduction in combined annual fuel and soil carbon retention-related emissions of conservation tillage, the share attributable to glyphosate is 30.6%. Additional information relating to these calculations for an example country (Argentina) is provided in Appendix 3.

## Discussion

### Total CO2e Emissions/Removals Associated with the Global Agricultural Use of Glyphosate

A summary of the total CO2e emissions/removals associated with the global agricultural use of glyphosate, inclusive of manufacture, distribution and farm level use of glyphosate and glyphosate’s estimated contribution to conservation tillage-related removals is presented in Table 12.

**Table 12.** Summary of global annual CO2e emissions/storage attributable to the use of glyphosate in agriculture: 2019–2022 annual average.

	Million Kgs CO2e	Car equivalents (‘000s)
Total CO2e emissions from farm level application of glyphosate in agricultural uses	1,368.5	906.9
Total CO2e emissions from manufacture and distribution of glyphosate to farmers	8,391.8	5,561.2
*Sum of total CO2e emissions from manufacture, distribution and application of glyphosate*	*9,760.3*	*6,468.0*
Total CO2e emissions from fuel use in conservation tillage balanced by soil carbon savings in conservation tillage area attributable to glyphosate	− 41,933.3 to −54,942.0 if this area had been plowed	− 27,788.8 to −36,409.5 if this area had been plowed
**Net total CO2e emissions**	**- 32,173 to −45,181.7**	**- 21,320.8 to −29,941.5**

Assumption: an average family car in 2024 produces 123.4 grams of carbon dioxide per km. A car does an average of 12,231 km/year and therefore produces 1,509 kg of carbon dioxide/year.

The net annual global impact of glyphosate use is providing a positive contribution to reducing global CO2e emissions arising from agricultural production of between −32.17 billion kg and −45.18 billion kg, equal to taking between 21.3 and 29.9 million cars off the road each year. The net annual global carbon storage with conservation tillage practices attributable to glyphosate is also equal to 30.6% of the total carbon storage associated with conservation tillage practices worldwide.

At an active ingredient level, the CO2e emissions associated with glyphosate manufacture, distribution and application by farmers is 13.03 kg CO2e per kg of active ingredient, balanced against a net CO2e storage associated with conservation tillage-related fuel savings and soil carbon sequestration attributable to glyphosate use of between −55.97 kg CO2e per kg of active ingredient and −73.33 kg CO2e of active ingredient, giving a net balance of between −42.94 kg CO2e per kg of active ingredient and −60.3 kg CO2e of active ingredient.

The point at which the conservation tillage-related fuel savings and soil carbon sequestration attributable to glyphosate cancels out the CO2e emissions associated with the manufacture, distribution and use of glyphosate is 13.03 Kg CO2e per kg of active ingredient. This is equal to between 5.4% and 7.1% of the total global combined annual fuel and soil carbon retention-related CO2e emissions saving from the global use of conservation tillage (presented in Table 6 and Table 7).

A recurring feature of the analysis is the dominance of the main grain and oilseed producing and exporting countries of North and South America in the adoption of conservation tillage practices and associated CO2e emission reduction as well as being the countries where the highest concentration of glyphosate use takes place. These countries account for 65% of global glyphosate use (in terms of active ingredient use), 72% of global CO2e emission reduction associated with conservation tillage practices and 79% of the share of these CO2e emissions reduction attributable to glyphosate use. As discussed in the results section, this dominance of the main grain and oilseed exporting countries of North and South American countries is also closely associated with the widespread adoption of GM HT crops tolerant to glyphosate. In the first 20 years of the widespread use of this technology, tolerance to glyphosate was the dominant trait and glyphosate application dominated weed control in these production systems largely because of its broad-spectrum post-emergence activity, ease of use and cost effectiveness. Often it was used as the sole method of weed control in the first few years of GM HT crop adoption. For example, glyphosate accounted for over 80% of total active ingredient use on GM HT soybean crops in the USA in the late 1990s/early 2000s (Brookes, 2022b^73^). As discussed earlier, GM HT (tolerant to glyphosate) technology also played a major role facilitating farmers adopting and staying in NT/RT-based conservation tillage production systems during this period.

This approach to weed control contributed to the evolution of weed populations predominated by resistant individual weeds and to weed shifts toward those weed species that are inherently not well controlled by glyphosate (Vencil et al., 2012^74^, Norsworthy et al., 2012^75^).

As a result, over the last 20 years, growers of GM HT crops have been using other herbicides (with different and complementary modes of action) in combination with glyphosate and in some cases adopting cultural practices (e.g., reverting to plowing) in more integrated weed management systems. In addition, GM HT crops tolerant to other herbicides (often stacked with glyphosate) have also become available from 2016 (notably to dicamba, 2 4 D and glufosinate). This has likely reduced the year-on-year absolute levels of CO2e emission reductions from NT/RT-based conservation tillage agriculture facilitated by GM HT crops relative to several years ago in North and South America (see for example Lu et al. (2022)^76^, Van Deynze et al. (2021)^77^). The estimates presented in this paper do, however, take this factor of influence into account by using the latest available data on the adoption of conservation tillage areas, the share of glyphosate use in the pre plant/burndown phase of crop production cycles and on the share of GM HT crops that are only tolerant to glyphosate as distinct from GM HT crops tolerant to glyphosate plus other herbicides.

### Sensitivity Analysis and Caveats to Analysis

Applying sensitivity analysis to the total emission assumption for the manufacture and distribution of glyphosate so that for example it was increased by an illustrative 20% to 13.44 kg of CO2e per kg of glyphosate active ingredient (to factor in the emissions associated with formulation of products containing glyphosate),^c^^c.^Not such data exists, hence presented for illustrative purposes. the total amount of annual CO2e emissions associated with the annual manufacture and distribution of glyphosate used in global agriculture would be 10.07 billion kg.

Applying sensitivity analysis to the calculations relating to the annual emissions associated with the farm level application of glyphosate shows that if the CO2e emissions are calculated around the main range of fuel used to apply herbicides around the world (0.5 to 1 liter of fuel per spray ha), the total amount of annual CO2e emissions associated with the application of glyphosate used in global agriculture is within the range of 0.82 billion kg and 1.63 billion kg. Combining the two elements of CO2e emissions associated with the manufacture, production and distribution of glyphosate with farm level application of products containing glyphosate, the total annual CO2e emissions is 9.76 billion kg, within a range of 9.21 billion kg and 11.7 billion kg.

Applying sensitivity analysis to the calculations of the amount of CO2e storage in the form of soil carbon arising from the use of conservation tillage practices facilitated by glyphosate is complicated by the many different factors of influence on soil carbon storage with conservation tillage practices. These include:

- The extent to which the hypothesis that soil organic carbon levels may peak after 20 years and then decline is applied to some areas reported to be using conservation tillage. However, this hypothesis is not widely accepted with other researchers such as Ussiri and Lal (2009^34^ arguing that very old soils continue to store carbon;
- What assumptions are made about how long the area (total or parts) of conservation tillage used to calculate the annual CO2e storage as soil carbon are assumed to have remained in permanent conservation tillage. A lack of comprehensive location-specific data makes this difficult to explore further although some analysis points to a range of 20% (Claassen R et al., 2018^78^ relating to the US combined area of corn, cotton, soybeans and wheat 2012-2016) and 90% (Argentine No-Till Farmers Association (AAPRESID)) cited in Brookes, 2022c^79^ relating to the main soybean, corn and wheat growing area in Argentina;
- The extent to which the use of NT/RT-based production conservation tillage systems also incorporate cover crops and operate crop rotation. There is also a lack of detailed data on these subjects. The only source identified in this research was CEAP^42^ relating to the USA, which estimated that in the 2013-2016 period there were 24.4 million hectares of arable land and 41.7 million ha in continuous NT (the area used for the USA in this analysis).

In addition, there is scope for applying sensitivity analysis to the assumed share of total CO2e stored as soil carbon with conservation tillage that is attributable to glyphosate depending on the criteria chosen. In this analysis, the share of the total herbicide spray area accounted for by glyphosate in the key phases of production (seed soil-bed preparation phase and pre-emergence) for the main crops in which conservation tillage is typically practiced was used.

This analysis has utilized several simplifying assumptions to calculate estimates of impact. The author also considers that the assumptions applied to the attributed share of glyphosate to conservation tillage-related CO2e emission/removals have been conservative. The research estimated that worldwide use of glyphosate annually contributes to a significant net CO2e emission reduction/removal through its facilitating role in the adoption and maintenance of NT/RT-based conservation tillage, which equates to the equivalent of nearly 31% of the total annual global conservation tillage-related CO2e emission savings/removals. Even if more conservative assumptions than those used in this study were applied, the share of the total annual global conservation tillage-related CO2e emission savings/removals attributed to glyphosate needs only be between 5.4% and 7.1% of these removals to cancel out the annual CO2e emissions associated with the manufacture, distribution and use of glyphosate.

Overall, there are many variables and different factors influencing each of these variables that could be included in analysis. To fully incorporate all possible variables that might simultaneously impact on soil carbon storage levels in crops grown using conservation tillage practices around the world (or even in a limited range of countries in North and South America) is likely to require a considerable input of resource and expertise across more than one discipline. It will also have considerable data requirements if more sophisticated analysis, such as building regional or farm level greenhouse gas emission calculator models were to be included. Some of this data is also unlikely to be available (e.g., comprehensive data on the area of remaining in conservation tillage in each country each year) and will therefore require the continued use of some simplifying assumptions.

All these factors should be taken into consideration if more sophisticated and complex analysis is to be undertaken beyond the findings of this work.

## Terminology

In the analysis and quantification of greenhouse gas (GHG) emissions associated with the production and use of inputs in agricultural production systems, GHG emissions may arise from the emission of several gases, of which the most common are carbon dioxide (CO2), methane (CH4) and nitrous oxide (N2O). As a specific unit of each gas (e.g., 1 kg) is not equivalent in terms of its greenhouse gas emission, comparisons are usually referred to in carbon dioxide equivalents (CO2e), with conversions into CO2e of a particular gas made using what is known as the global warming potential (GWP) of each gas. For example, the GWP of methane (CH4) is 27.9 meaning that 1 kg of methane emitted is the equivalent of 27.9 kg of carbon dioxide. In this paper, all references to carbon dioxide emissions are in terms of carbon dioxide equivalence (CO2e).

## Appendix 1 Conservation Tillage Impacts on Carbon Storage: Summary of Main Literature Reviewed.

Soil organic carbon can be depleted through the long-term application of poor farming practices and the conversion of natural eco-systems (such as forest, prairie and steppes) into crop and grazing land. These agriculture-related uses deplete the soil organic carbon pool by increasing the rate of conversion of soil organic matter to carbon dioxide, thereby reducing the input of biomass carbon and accentuating losses by erosion. The significant degradation of crop soils by the oxidation of soil carbon into carbon dioxide started in the 1850’s with the introduction of large-scale soil cultivation using the plow. Tillage was the preferred form of cropping agriculture because it loosened soils making planting easier, aided the addition of vegetation and nutrients, the removal of a crust, reduction of compaction, aeration and as a part of weed control. However, tillage also resulted in some serious problems, especially soil erosion and degradation. Pimental et al (1995^98^) estimated that about 430 million hectares of arable land, equal to about a third of the global total had been lost by the mid-1990s to soil erosion. In addition, Lal et al (1999^69^) estimated that the global release of soil carbon since 1850 from land use changes has been 136 +/- 55 Pg^d^ ^d.^ Pg of soil carbon pool equates to 0.47 parts per million of atmospheric carbon dioxide. (billion tonnes) of carbon. This is approximately half of the total carbon emissions from fossil fuels (270 +/- 30 Pg (billion tonnes)), with soil cultivation accounting for 78 +/- 12 Pg and soil erosion 26 +/- 9 Pg of carbon emissions. Lal et al (1999^69^) also estimated that the potential of carbon sequestration in soil, biota and terrestrial ecosystems may be as much as 3 Pg C per year (1.41 parts per million of atmospheric carbon dioxide). The authors stated “reversing these trends can be achieved by a variety of soil and crop management technologies and approaches that increase soil organic carbon storage through the removal of atmospheric carbon dioxide. These include:

- The adoption of no-till farming with residue mulch and cover cropping;
- integrated nutrient management (INM), which balances nutrient application with use of organic manures and inorganic fertilizers;
- various crop rotations (including agro-forestry);
- use of soil amendments (such as zeolites, biochar, or compost); and improved pastures with recommended stocking rates and controlled fire as a rejuvenate method (Lal (2010^13^)).”

In sub-humid and semi-arid regions, switching from conventional tillage to conservation tillage also accompanied by a reduction in summer fallow or intensification of cropping systems often result in additional soil carbon sequestration (McConkey et al, 2003^99^). The production benefits of increasing soil carbon storage also include increased soil infiltration, fertility and nutrient cycling, decreased wind and water erosion, minimal soil compaction, enhanced water quality, decreased carbon emissions, impeding pesticide movement and generally enhanced environmental quality. Quantification of the impacts of tillage on carbon stocks is complex due to the combination and complexities of soil, climate and management conditions, especially crop type and rotation. Issues affecting the levels of carbon sequestration include:

- Soil and climatic factors;
- The depth examined/sampled – for example, limiting sampling to relatively shallow depths may overstate carbon sequestration level in NT systems;
- Initial soil carbon levels;
- Crop biomass production (soil carbon inputs) including roots and associated mycorrhizea;
- Organic carbon mineralization (soil carbon outputs); and
- Soil erosion and re-deposition on soil organic gains and losses.

Nevertheless, there is general agreement that the potential for sequestration or storage of carbon in soil is significant. Zomer et al (2017^100^) estimated that globally crops could possibly sequester between 0.90 and 1.85 Pg C/yr, equivalent to 26-53% of the target of the “4p1000 Initiative: Soils for Food Security and Climate”^e^ ^e.^ 4p1000 - The aim of the initiative is to demonstrate that agriculture, and in particular agricultural soils can play a crucial role where food security and climate change are concerned. https://www.4p1000.org/. However, there are limitations to achieving the full potential by location and within specific farming systems, including a lack of biomass and other inputs. As a result, efforts to sequester carbon should, in the view of some analysts (e.g., Van Groenigen et al., 2017^101^), be concentrated on soils that have become degraded due to long periods of intensive arable cropping in temperate climatic regions in Asia, Europe and North America. Many researchers have examined issues relating to carbon sequestration and different tillage systems (see table in separate file).

**Table A1.** Conservation tillage impacts on carbon storage: summary of main literature reviewed.

Reference	Type of study	Key findings
Buragiene et al (2019^16^)	Site-specific experimentation research (Lithuania)	Reviewed different tillage technologies and their effect on carbon dioxide emissions from the soil. Concluded that deep plowing immediately increases carbon dioxide emissions up to seven times higher that NT
Mangalassery et al (2014^17^)	Sample based collection of data from arable farms in the UK	Analyzed the greenhouse gas (GHG) balance for the carbon dioxide, methane and nitrous oxide emissions for conventional tillage and zero tillage systems. This research concluded that the net global warming potential under conventional tillage systems was 26%-31% higher than zero tillage. Although nitrous oxide emissions increased under zero tillage this was counter-balanced by a significant reduction in potential carbon dioxide and methane emissions
Nicoloso and Rice (2019^18^)	Meta-analysis	Assessed carbon and nitrogen storage and sequestration in no-till soils from the most important agricultural regions of the world. Identified that NT soils store both **more** carbon and nitrogen (up to 100 cm depth) than tilled soils. However, carbon sequestration depended on; an increase in crop frequency (eg. the rapid replanting of arable land following harvest); additional nitrogen inputs applied to each crop to ensure crop establishment; and decreased soil disturbance. Single cropping with land left fallow between harvesting and reseeding lacks carbon inputs to maintain soil carbon throughout the soil profile. The use of legumes alleviates nitrogen losses and supply extra nutrient to support carbon sequestration. Findings indicate that no-till can effectively mitigate climate change by either avoiding CO_2_ emissions from tilled soils or by promoting soil carbon sequestration in intensified agricultural systems
West and Marland (2003^80^)	Site-specific experimentation research (USA)	Estimated that the net carbon flux from the conversion from CT to NT was a decrease of 468 kg/C ha^−1^yr^1^ for maize, 32 kg/C ha^−1^yr^1^ for wheat and 371 kg C ha^−1^yr^1^ for soybeans released to the atmosphere
West and Post (2002^68^)	Analyzed 67 long-term agricultural experiments, consisting of 276 paired treatments	Results indicate, on average, that a change from CT to NT can sequester 57 ± 14 g carbon per square meter per year (grams carbon m^−2^ year^−1^), excluding a change to NT in wheat-fallow systems. The cropping system that obtained the highest level of carbon sequestration when tillage changed from CT to NT was maize – soybeans in rotation (90 ± 59 grams carbon m^−2^ year^−1^). This level of carbon sequestration equates to 900 ± 590 kg/C ha^−1^yr^1^, which would have decreased carbon dioxide level in the atmosphere by 3,303 ± 2,165 kg of carbon dioxide ha^−1^yr^1^
Ogle et al (2019^22^)	Meta-analysis	Reviewed the impact of CT compared with NT in different climatic environments. They found that converting from CT to NT over a twenty-year period resulted in an increase in SOC storage of 23% in tropical moist climates, 17% in tropical dry climates, 16% in temperate moist and 10% in dry climatic conditions
Huggins et al (2007^81^)	Sample based collection of data from farms and experimental sites in the USA	Assessed over a 14-year period crop sequence and tillage effect on SOC dynamics and storage, in continuous maize or soybeans and alternating maize-soybeans under different tillage treatments. CT soybeans and fallow decreased SOC at an average annual loss of 3.7 Mg/C ha^−1^yr^1^, while chisel plough (RT) with continuous maize or maize-soybeans and NT with continuous maize, averaged an annual loss of 1.6 Mg/C ha^−1^yr^1^. Concluded that without large additional carbon inputs (eg, manures, cover crops, perennial crops) the potential to approach SOC levels of native sites is limited with annual cropping and RT
Johnson et al (2005^6^)	Literature review	Summarized how alternative tillage and cropping systems interact to sequester soil organic carbon (SOC) and impact on GHG emissions from the main agricultural area in central USA. This analysis estimated that the rate of SOC storage in NT compared to CT has been significant, but variable, averaging 400 ± 61 kg/C ha^−1^yr^1^
Calegari et al (2008^7^)	Site-specific experimentation research (Brazil)	Conducted a 19-year experiment comparing CT and NT management systems with various winter cover crop treatments in Brazil. The research identified that the NT system led to 64.6% more carbon being retained in the upper soil layer than in the CT system. It also found that using NT with winter cover crops resulted in soil properties that most closely resembled an undisturbed forest (ie, best suited for greenhouse gas storage). In addition, both maize and soybean yields were found to be respectively 6% and 5% higher, under NT, than CT production systems
Eagle et al (2012^82^)	Literature review	Examined the literature on GHG mitigation potential of conservation tillage and NT. Based on 280 field comparisons of soil carbon response to NT the average mitigation potential was estimated at 327 C ha^−1^yr^1,^ equal to 1,200 kg of carbon dioxide ha^−1^yr^1^ with a range of −200 to 3,200 ha^−1^yr^1^
Olson et al (2013^83^)	Site-specific experimentation research (USA)	Evaluated soil carbon levels over a 24-year period on eroded soils in Southern Illinois that were under a maize and soybeans rotation that used different tillage systems. The NT system stored and retained 7.8 tonnes of carbon per ha more than CT plots (equal to 325 C ha^−1^yr^1^)
Kahlon et al (2013^84^)	Site-specific experimentation research (USA)	Evaluated different tillage practices and the importance of mulching on soil physical properties and carbon sequestration over a period of 22 years. The NT plots consistently resulted in positive effects on soil physical attributes and total carbon concentration
Haruna and Nkongolo (2019^19^)	Site-specific experimentation research (USA)	Reviewed the rate of change in soil organic matter (SOM) for maize and soybeans with and without cover crops under CT vs NT. NT resulted in 4% higher SOM and 8% higher SOM with a cover crop
Bernoux et al (2006^85^)	Literature review	Reviewed cropping systems, carbon sequestration and erosion in Brazil. Found over 30 years of NT practice carbon levels in topsoil increased. Identified the rate of carbon storage in the top 40 cm of the soil ranges from 400 to 1,700 kg C ha^−1^yr^1^ in the Cerrado region. The mean rates of carbon storage in the soil surface area (0–20 cm) varied from 600 to 680 kg C ha^−1^yr^1^ with the greatest variation in the southern region of −70 to 1,600 kg C ha^−1^yr^1^ (standard deviation 680 ± 540 kg C ha^−1^yr^1^). In addition, in Brazilian conditions direct seeding offers the scope for earlier sowing of crops, shortening the total production cycle, facilitating a second crop in the same season. This results in more carbon being returned to the soil
IPCC (2006^4^)	Expert panel review of literature	Estimated the rate of soil organic carbon (SOC) sequestration by the conversion from conventional to all conservation tillage (NT and RT) in North America within a range of 50 to 1,300 kg C ha^−1^yr^1^ (it varies by soil type, cropping system and eco-region), with a mean of 300 kg C ha^−1^yr^1^
Robertson et al (2000^5^ and Sexstone et al (1985^86^)	Sample based collection of data from farms and experimental sites in the USA	Findings suggested that the adoption of NT (sequestering SOC) could do so at the expense of increased nitrous oxide production if growers were to increase the use of nitrogen fertilizer in NT production systems
Almaraz (2009^68,87^)	Sample based collection of data from farms and experimental sites in Canada	Examined the importance of nitrogen fixing legume grain crops by studying the GHG emission associated with N_2_ fixing soybean grown under CT and NT tillage systems. Findings suggest that using NT in N-fixing legume crops may reduce both carbon dioxide and N_2_O emissions in comparison to CT, because in the CT system, harvest residue is incorporated into the soil during plowing (increasing N_2_O emissions)
Omonode et al (2011^88^)	Site-specific experimentation research (USA)	Assessed N_2_O emissions in maize following three decades of different tillage and rotation systems. Seasonal cumulative N_2_O emissions were significantly lower by 40%-57% under NT compared to long term chisel and moldboard plow tillage systems, due to soil organic C decomposition associated with higher levels of soil residue mixing and higher soil temperatures
Johnson et al (2005^6^)	Research analysis	Using IPCC emission factors, estimated the offsetting effect of alternative fertilizer management and cropping systems. For a NT cropping system that received 100 kg N per ha per year (net from all sources), the estimated annual nitrous oxide emission of 2.25 kg N per ha per year would have to increase by 32%-97% to completely offset carbon sequestration gains of 100–300 kg per ha per year
Baker et al (2007^8^)	Meta-analysis	Identified 37 out of 45 studies (from 17 experiments) with sampling depth < 30 cm at which NT treatments (82%) reported more SOC than in the CT control with a mean annual SOC gain of 380 ± 720 kg C ha^−1^yr^1^. In contrast, in 35 of 51 studies (from 5 experiments) with sampling depths > 30 cm, the NT treatments registered less SOC relative to CT with a mean annual loss of −230 ± 970 kg C ha^−1^yr^1^. This work questioned the premise that NT leads to positive carbon sequestration compared to CT. In both cases, however, the standard error associated with the estimates was so large that the mean (impact of tillage) was not considered to be significant
Angers and Eriksen-Hamel (2008^9^)	Literature review	This work compared NT and full-inversion tillage (FIT) trials and found that while there was a statistically significant increase in total SOC stocks under NT (100.3 versus 95.4 Mg C ha^−1^ for NT and FIT respectively in the upper 10 cm), to the 21–25 cm soil depth (which corresponds to the mean plowing depth (23 cm)), the average SOC content was significantly greater under FIT than NT. It was also greater under FIT just below the average depth of plowing (26–35 cm). However, there was significantly more SOC (4.9 Mg ha^−1^) under NT than FIT across all depths and this difference in favor of NT increased weakly with the duration of the experiment
Blanco-Canqui & Lal R (2007^10^).	Site-specific experimentation research (USA)	This research found that the majority of SOC increase under NT is in the top 10 to 15 cm of soil with insignificant changes (or even decreases) in SOC relative to CT at depths over 15 cm. Hence, newly sequestered carbon in a NT system is accumulated where it is most vulnerable to environmental and management pressures. This makes any permanent increase in SOC associated with NT systems vulnerable to changes in environmental pressures and soil management practices
Syswerda et al (2011^89^)	Site-specific experimentation research (USA)	This study examined whether soil sequestration gains in the surface layer may result in soils losing carbon at depth under NT compared with CT. Results indicated that surface soil carbon concentrations and total carbon pools were significantly greater under NT than CT. No difference in soil carbon at depth was identified although carbon levels were found to be variable. It also concluded that there was no evidence of carbon gains in the surface soils of NT being either offset or magnified at depth
Al-Kaisi M (2005^90^)	Sample based collection of data from farms and experimental sites in the USA	Evaluated the effects of different tillage systems on soil organic carbon (SOC) and nitrogen (SON), residue carbon and nitrogen inputs and crop (maize and soybean) yields in Iowa. Yields of both maize and soybean were comparable in NT and moldboard tillage systems but in NT and strip-tillage there was a significant increase in SOC of 14.7% and 11.4% respectively. Changes in SON due to tillage were like those observed with the SOC experiments
Hollinger et al (2005^91^)	Research analysis and modelling	This study measured the carbon budget for maize and soybean in rotation that had been in NT cultivation for over 14 years. The carbon sink when planted with maize was 576 g C m^−2^ per year and soybean 33 g C m^−2^ per year. Accounting for 100% grain consumption, maize acts as a C-sink of 184 g C m^−2^ per year while soybean becomes a C-source of 94 g C m^−2^ per year. As these crops are generally grown in rotation, this system is a net sink of 90 g C m^−2^ per year
Clapperton (2003)^92^	Research analysis	Identified that NT soils are more biologically active and diverse, have higher nutrient loading capacities, release nutrients gradually and continuously and have better soil structure than reduced or cultivated soils. By enhancing the organic matter, a higher Carbon-Stock Equilibrium (CSE) can be achieved
Lal R (2010^13^).	Research analysis	Identified that the rate of soil carbon sequestration through the adoption of NT/RT management practices on degraded soils ranges from 100 kg C ha^−1^yr^1^ in warm and dry regions to 1,500 kg C ha^−1^yr^1^ in cool and temperate regions. It also estimated the technical potential of soil organic carbon sequestration through adoption of RMPs for world cropland soils (1.5 billion ha) to be 0.6 billion to 1.2 billion tonnes of carbon per year and about 3 billion tonnes of carbon per year in soils of all ecosystems (eg, cropland, grazing land, forest lands, degraded lands and wetlands
Conant et al (2007^93^), Venterea et al (2006^94^)	Literature review and analysis	These studies identified that intermittent tillage during long‐term RT or NT is needed to reduce soil compaction, for weed control, or to reduce pests or pathogens. Whilst, intermittent tillage can cause a decrease in soil stocks, up to 80% of soil gains from NT practices can be maintained when implementing NT with intermittent tillage
Walia et al (2017^95^)	Site-specific experimentation research (USA)	This examined in southern Illinois the tillage and fertilizer use effects on bulk density and soil carbon concentrations over a 44-year period (20 years in continuous maize and 24 years in maize – soybean rotation). NT management increased carbon stocks compared to tillage for depths of between 0 to 15 cm. NT combined with NPK (nitrogen, phosphorus and potassium) fertilizer maintained greater cumulative soil carbon stocks to 1 meter soil depth than either undisturbed forest soils or restored prairie soils. Additionally, NT/NPK had a maximum soil carbon increase over time of 360 kg C ha^−1^yr^1^ for the top 15 cm over 44 years
Virto et al (2012^96^)). Yang et al (2013^97^)	Research analysis and modelling	These studies questioned the accuracy, and the level of carbon sequestered previously projected for NT compared with CT. The studies concluded that it may not be appropriate to attribute all the higher carbon content in the surface of NT soil to either increased carbon input or reduced carbon mineralization (output) relative to CT, when the differences may be due to soil erosion
Powlson et al (2014^3^)	Literature review	Questioned the assumptions of the UN Emissions Gap Report 2013 which presented a case that additional adoption of NT could further contribute to more carbon sequestration because much of the most suitable land for adoption of NT is already using this production system. The study did, however, acknowledge that widespread adoption of NT in North and South America had delivered important carbon sequestration savings and if this land was to revert to CT, it would result in significant carbon release
Olge et al (2019^22^)	Literature review	This explored literature relating to the adoption of NT management, carbon storage and the 4 per mille (4p1000) initiative promoted through the UN Framework Convention on Climate Change. The research concluded that SOC storage can be higher under NT management in some soil types and climatic conditions even with redistribution of SOC and contribute to reducing net greenhouse gas emissions. However, *uncertainties* tend to make this approach less attractive as a contributor to *stabilise* the climate system compared to other options. This research concluded that the adoption of NT may be better viewed as a method of reducing soil erosion, adapting to climate change, ensuring food security and valuing any increase in SOC storage as a “co-benefit^f^” for society in terms of reducing greenhouse gas emission
Haddaway et al (2017^20^)	Meta-analysis	This large-scale systematic review of research examining the impact of different tillage systems on soil organic carbon levels. It reviewed a total of 351 studies and found that soil organic concentrations were significantly higher in no tillage relative to both reduced tillage and conventional tillage in the upper soil layer (up to 30 cm depth). This was equal to difference of 460.6 kg C ha^−1^yr^1^ over a period greater than 10 years (based on 29 studies). Evidence of differences across a deeper soil profile (0 cm to between 30 cm and 150 cm depth, the evidence from 14 studies was less conclusive and not overall statistically significant difference was found
Maraseni and Cockfield (2011^38^)	Research analysis	This study examined the impact of the adoption of zero tillage on dryland tillage systems in the Darling Downs region of Queensland. In aggregate zero tillage winter crops release less Co2 than conventional tillage crops, though this depends on location and type of farming. It concluded that zero tillage increases levels of soil carbon in upper soil layers and down to 20 cm but queried if it continues below this level
Abdulla et al (2016^25^)	Meta-analysis	This research analyzed 46 peer reviewed papers totaling 174 paired observations that compared CO2 emissions from tilled and untilled soils across different climates, crop types and soil conditions. The analysis concluded that that on average tilled soils emitted 21% more CO2 than untilled soils on a worldwide basis. This difference was greatest (29%) in sandy soils in arid climates with low soil organic carbon content and low soil moisture. In contrast, they found little difference in clay soils with high background soil organic carbon levels. Crop rotation allied to no till practices increased the difference to 26% relative to tilled soils, with the research emphasizing the importance of including soil factors such as texture, aggregate stability and organic carbon content in global models of the carbon cycle
Ussiri and Lal (2009^34^)	Site-specific experimentation research (USA)	This research observed increases in SOC in the top 30 cm of soil of 1.37 Mg C ha-1 year-1 (1,370 kg of carbon per ha per year) after 43 years of continuous maize under no tillage. This compared with a rate of 0.73 Mg C ha-1 year-1 (730 kg C ha^−1^yr^1^) for continuous maize under conventional tillage
Liang B et al (2020^26^)	Meta-analysis	Updated earlier analysis from 2007 and reviewed 36 studies undertaken since 1997 into conservation practices in Canada. The analysis identified an average increase in SOC with no till equal to 211 kg C ha^−1^yr^1^ compared to decrease in SOC of 10 kg C ha^−1^yr^1^ for tillage across a mix of soils (coarse, medium and fine) both in the West and East of the country. The net impact of using NT practices was −201 kg C ha^−1^yr^1^ equal to 774.37 kg of co2 decreased per ha with NT
Hasson W et al (2022^21^)	Meta-analysis	This research reviewed zero tillage impacts on greenhouse gas emissions. It summarized the findings from 22 studies with differences between co2 emissions with zero tillage relative to conventional tillage of between 1.2% and 75% (3 over 50% difference, 9 with differences between 25% and 50%, 7 with differences in the range −10% to −25% and the rest (3) under 10%)
Mazzoncini M et al (2016^24^)	Long term experiment	This long-term experiment in Central Italy examined SOC with NT and CT. It identified that SOC levels were higher in NT than CT (1986–2014) in the soil layer 0-30 cm (in 2014 Conventional tillage SOC was 16.46 Mg C-ha compared to 27.79 Mg C-ha for NT – a rise of 22% in NT from base of 22.78 or in terms of g C 100 g-1 NT 1.96 in 2014 1.59 in 1996 and CT 1.12 in 1996 and 1.06 in 2014. This provides a mean rate of soil C sequestration of (27.8–16.5)/28 = 404 kg C ha^−1^yr^−1^
Perry et al (2016^49^)	Review of literature	Reviewed papers for evidence of a relationship (complementarity) between the adoption and use of glyphosate tolerant crops and conservation tillage practices. It concluded that the evidence in favor of complementarity between glyphosate tolerant crops and conservation tillage outweighs the evidence against it. The research further examined the relationship using farm level data from the US for nearly 30,000 soybean farmers over the period 1998–2011 and confirmed complementarity, with adoption rates for CT and NT estimated to have been 10% and 20% respectively higher than would otherwise have occurred in the absence of glyphosate tolerant soybean technology

^f^Co-benefits of climate change mitigation as defined in the 4th Assessment Report of the Intergovernmental Panel on Climate Change are the positive benefits related to the reduction of greenhouse gases.

## Appendix 2

**Table A2.** Estimated CO2e emissions from the manufacture, distribution and application of glyphosate in agriculture by country where use takes place: annual average based on 2019-2022 usage data.

Country	Energy-related emissions derived from the manufacture and distribution of the volumes of glyphosate used by farmers in each country (kgs CO2e)	Fuel-related emissions from application of glyphosate (kgs CO2e)
Algeria	666,655	53,091
Angola	967,680	168,000
Argentina	1,304,464,110	280,749,280
Australia	309,250,189	87,182,171
Austria	640,044	132,108
Bangladesh	3,387,328	106,496
Belarus	18,882,959	2,457,669
Belgium	402,850	93,339
Benin	7,336,759	686,724
Bolivia	97,216,560	11,998,560
Brazil	1,831,586,820	280,002,318
Bulgaria	423,685	55,162
Burkina Faso	5,147,120	342,576
Cameroon	14,934,640	1,087,299
Canada	350,965,188	78,938,221
Central America-Caribbean	1,194,984	127,749
Chile	24,572,509	2,922,270
China	744,903,807	93,022,721
Colombia	60,626,762	5,830,611
Costa Rica	2,353,820	332,962
Côte d’Ivoire	90,424,480	7,245,066
Croatia	856,094	146,661
Cuba	4,601,223	427,953
Czech Republic	1,078,602	203,840
Denmark	12,552,257	6,499,432
Dominican Republic	4,505,007	360,146
Ecuador	32,385,511	7,106,256
Egypt	8,262,304	590,321
El Salvador	2,801,095	392,650
Estonia	621,656	114,110
Ethiopia	10,303,574	2,368,800
Finland	7,074,043	5,983,807
France	33,392,150	7,780,771
Germany	40,962,603	8,672,307
Ghana	26,610,105	1,611,482
Greece	15,315,241	1,628,202
Guatemala	5,746,118	887,933
Honduras	3,596,835	504,694
Hungary	5,485,312	646,565
India	130,593,800	4,060,846
Indonesia	126,776,742	12,357,143
Ireland	200,880	52,156
Israel	9,393,952	987,166
Italy	32,060,577	5,273,656
Japan	73,638,270	16,662,961
Kazakhstan	60,046,241	22,224,679
Kenya	6,337,464	869,367
South Korea	29,881,118	3,229,245
Latvia	199,444	42,586
Lithuania	275,352	46,960
Malawi	10,761,666	609,951
Malaysia	48,694,744	9,511,693
Mali	20,085,232	2,692,579
Mexico	55,262,841	9,782,154
Moldova	409,534	70,609
Morocco	872,502	124,157
Mozambique	22,182,026	2,199,278
Myanmar	354,659	23,991
Namibia	21,235	2,359
Netherlands	5,861,570	916,483
New Zealand	16,613,649	2,611,404
Nicaragua	3,075,313	167,162
Norway	5,252,713	2,620,833
Pakistan	8,947,896	223,697
Panama	2,434,617	205,543
Paraguay	148,571,270	18,930,688
Peru	9,591,162	1,859,054
Philippines	23,232,490	2,451,240
Poland	3,342,354	631,029
Portugal	11,997,882	1,556,952
Romania	5,192,922	887,707
Russia	121,076,976	21,053,316
Senegal	2,079,694	277,647
Serbia	10,609,435	1,195,320
Slovakia	993,409	146,630
Slovenia	495,790	43,660
South Africa	557,805,497	16,421,458
Spain	44,602,309	8,855,310
Sweden	10,378,382	4,789,063
Switzerland	898,825	151,370
Taiwan	6,168,820	761,078
Tanzania	9,168,021	634,667
Thailand	132,538,361	12,555,191
Tunisia	4,549,415	337,178
Turkey	14,523,880	2,674,883
Ukraine	15,454,835	2,403,740
United Kingdom	21,098,557	4,670,938
United States	1,397,488,856	258,662,194
Uruguay	52,177,818	7,650,460
Venezuela	19,350,349	1,078,242
Vietnam	3,250,666	72,112
Zambia	4,527,849	331,945
Zimbabwe	3,894,719	381,933
Total	8,391,793,263	1,368,493,982

Note: The emissions from the manufacture and distribution of glyphosate stated for each country are based on applying the global average 11.2 kg of CO2e emissions per kg active ingredient of glyphosate manufactured and distributed to farm level (source: Ecoinvent) to the total amount of glyphosate active ingredient used in agriculture in each country. Glyphosate manufacture does not take place in each country and a significant volume of glyphosate is traded between countries. The emissions values from the manufacture and distribution per country therefore reflect farm level usage not where the manufacture of glyphosate takes place.

## Appendix 3

**Table A3.** Example calculations of emissions: Argentina.

Row	Category	Values	Comments/source
1	Annual application of glyphosate (spray area ‘000 ha)	125,335	Sources: Private market research companies (Kleffmann, Kynetec), industry estimates, national import/export statistics, author own calculations
2	Assumed co2e emitted per ha sprayed: kg co2e/ha	2.24	Lazarus, 2019
3	Aggregate emissions from spraying glyphosate (kg co2e)	280,749,280	Row 1 x Row 2
4	Annual amount of glyphosate used in agriculture (‘000 kg active ingredient)	116,470	Sources: Private market research companies (Kleffmann, Kynetec), industry estimates, national import/export statistics, author own calculations
5	Assumed co2e emitted per kg active ingredient: kg co2e/ha	11.2	Source: Ecoinvent
6	Aggregate emissions from manufacture and distribution of glyphosate (kg co2e)	1,304,464,110	Row 4 x Row 5
7	Conservation tillage area (‘000 ha)	32,870	Kassam et al, 2022
8	Assumption for co2e kg emitted ha from fuel use with conservation tillage	58.45	See Table 2
9	Assumption for co2e kg emitted ha from fuel use with plough-based tillage	130.86	See Table 2
10	Aggregate fuel related co2e emissions on conservation tillage area (kg co2e)	1,923,414,150	Row 7 x Row 8
11	Aggregate fuel related co2e emissions if conservation tillage area was ploughed (kg co2e)	4,306,210,020	Row 7 x Row 9
12	Net emission savings from fuel use with conservation tillage compared fuel use if this land area was ploughed	2,382,795,870	Row 11 – Row 10
13	Assumption for co2e kg emission/ha from soil carbon sequestration with conservation tillage (-ve sign denotes a saving from carbon storage in the soil)	-642.25	Alvarez et al, 2014
14	Assumption for co2e kg emitted/ha from soil with plough-based tillage	90.75	Alvarez et al, 2014
15	Aggregate soil carbon related co2e emissions on conservation tillage area (kg co2e)	-21,134,520,750	Row 7 x Row 13
16	Aggregate soil carbon related co2e emissions if conservation tillage area was ploughed (kg co2e)	3,019,217,250	Row 7 x Row 14
17	Net emission savings from soil carbon related emissions with conservation tillage compared soil carbon related emissions if this land area was ploughed (kg co2e)	-24,153,738,000	Row 15 + Row 16
18	Aggregate fuel use and soil carbon related co2e emissions on conservation tillage area (kg co2e)	-19,211,106,600	Row 10 + Row 15
19	Aggregate fuel use and soil carbon related co2e emissions on conservation tillage area if ploughed (kg co2e)	7,325,427,270	Row 11 + Row 16
20	Net emission saving from conservation tillage compared to if this area was ploughed	-26,536,533,870	Row 18 + Row 19
21	Annual area of glyphosate sprayed in pre plant/pre-emergence phase (important for conservation tillage facilitation) in crops using conservation tillage (‘000 ha)	34,598	Sources: Private market research companies (Kleffmann, Kynetec), industry estimates, national import/export statistics, author own calculations
22	% of total glyphosate area sprayed used in pre plant/pre-emergence phases	29%	
23	Aggregate fuel use and soil carbon related co2e emissions on conservation tillage area attributable to glyphosate (kg co2e)	-5,577,591,507	Row 18 x Row 22
24	Aggregate fuel use and soil carbon related co2e emissions on conservation tillage area if ploughed attributable to glyphosate (kg co2e)	2,126,803,092	Row 19 x Row 22
25	Net emission saving from conservation tillage compared to if this area was ploughed attributable to glyphosate	-7,704,394,599	Row 23 + Row 24
26	Total emissions associated with glyphosate use inclusive of emissions from mfg, distribution, application plus net emission saving from conservation tillage compared to if this area was ploughed attributable to glyphosate	-6,119,181,210	Row 25 + Row 6 and Row 3
